# Surface Evaluation of Gyroid Structures for Manufacturing Rubber–Textile Conveyor Belt Carcasses Using Micro-CT

**DOI:** 10.3390/polym16010048

**Published:** 2023-12-22

**Authors:** Jozef Tkac, Teodor Toth, Gabriel Fedorko, Vieroslav Molnar, Miroslav Dovica, Sylwester Samborski

**Affiliations:** 1 Faculty of Manufacturing Technologies, Technical University of Kosice with a seat in Presov, Sturova 31, 08001 Presov, Slovakia; jozef.tkac@tuke.sk; 2 Faculty of Mechanical Engineering, Technical University of Kosice, Letna 9, 04200 Kosice, Slovakia; teodor.toth@tuke.sk (T.T.); miroslav.dovica@tuke.sk (M.D.); 3 Faculty of Mining, Ecology, Process Control and Geotechnology, Technical University of Kosice, Park Komenskeho 14, 04200 Kosice, Slovakia; gabriel.fedorko@tuke.sk; 4 Department of Fundamentals of Production Engineering, Faculty of Mechanical Engineering, Lublin University of Technology, Nadbystrzycka 36, 20-618 Lublin, Poland; s.samborski@pollub.pl

**Keywords:** carcass, additive manufacturing, rubber–textile conveyor belt, gyroid, CT scanning

## Abstract

Gyroid structures are among the most widely used three-dimensional elements produced by various additive manufacturing technologies. This paper focuses on a metrological analysis of Flexfill 92A material specimens with a relative density (25 to 85%) using industrial computer tomography. The results show that for a given structure, the best method is to use surface determination with the closure of internal defects in the material. The analysis implies that the smallest deviations of the specimens’ external dimensions were achieved with respect to the CAD model at the highest relative densities. The wall thickness shows the smallest percentage change of 0.5685 at 45% relative density and the largest at 25% and 85% relative density. The nominal–actual comparison of manufactured specimens to the CAD model shows the smallest cumulative deviation of 0.209 mm at 90% and 25% relative density, while it slightly increases with increasing relative density. All produced specimens have a smaller material volume than their theoretical volume value, while the percentage change in volume is up to 8.6%. The surface of specimens is larger compared with the theoretical values and the percentage change reaches up to 25.3%. The percentage of pores in the specimens increases with increasing relative density and reaches 6%. The acquired knowledge will be applied in the framework of research focused on the possibilities of using additive manufacturing to produce a skeleton of rubber–textile conveyor belts. This paper presents initial research on the possibility of replacing the carcass of rubber–textile belts with an additive technology use.

## 1. Introduction

The use of rubber–textile conveyor belts for the continuous transport of materials dates back to the 19th century [1]. Their subsequent deployment and use have gradually increased, rendering them important components of various transport systems in several industries [2].

Over the years, the construction and production of durable and reliable rubber–textile conveyor belts have gradually developed to facilitate efficient transport performance [3]. Typically, the production process of rubber–textile conveyor belts can be divided into the production of mixtures and tensile materials, which are primarily textile layers. Highly specialized production equipment has been developed for individual processes in the production of rubber–textile conveyor belts. Depending on the design of the belt, each step enables a discontinuous or continuous production process [4]. At present, the production of conveyor belts occurs in sophisticated production lines [5].

The advent of Industry 4.0 has led to the emergence of novel technologies, which are significantly changing and modernizing the characteristics of conveyor belts and belt transport systems [6]. The trend was primarily observed in the operation of belt conveyors, indicating that they require effective monitoring [7]. The monitoring of belt transport systems focuses on the detection of various errors, primarily associated with conveyor belts. However, other operational characteristics of the belt conveyors also require monitoring [8]. Therefore, progressive approaches based on modern technologies, such as machine vision [9], audio–visual fusion [10], and laser technology [11], are being deployed.

Furthermore, Industry 4.0 has affected the construction and production of rubber–textile conveyor belts. Industry 4.0 facilitates the implementation of several progressive technologies in engineering and industrial practices with additive technologies maintaining a dominant position. The extent of the development of additive technologies and their potential generates certain prerequisites for their partial or complete use in the production of rubber–textile conveyor belts and their structural parts. Based on this knowledge, the utilization of additive technologies in the production of rubber–textile conveyor belt carcasses, comprising industrial textiles connected via vulcanization [12,13,14], appears to be a prospective area of analysis.

Textile carcasses for conveyor belts have been extensively researched from a material perspective [15,16]. Therefore, suitable materials should be identified for use in additive technologies to produce textile carcasses for conveyor belts. At present, different types of materials are commercially available with the potential to be applied in the additive production of rubber–textile conveyor belt carcasses [17,18]. The ability of existing additive technologies to produce a structure that can outperform traditional conveyor belt carcasses should be verified before further analysis. Therefore, the objective of this study was to verify the feasibility of producing a rubber–textile conveyor belt using additive technology. The application of gyroids for the production of the carcass material was examined with consideration for the internal and external geometries of the fabricated specimens. The study findings form the basis for analyzing the material characteristics of carcasses that can affect the performance of conveyor belts.

## 2. Materials and Methods

Additive technologies are widely deployed in several industries to replace conventional production technologies. They can increase production, ensure the high quality of products, reduce production costs, and enable the production of products that cannot otherwise be manufactured using conventional technologies.

Although research on the use of additive technologies is constantly progressing, their use is limited by the existing technological and material possibilities. However, this does not affect their possible future applications.

From an application perspective, conveyor belt production is an area where additive technologies are rarely used; the production of conveyor belts has followed conventional processes since their inception. To date, innovations in the production or construction of conveyor belts have only considered technological conditions in production and the degree of automation. However, with the progress of additive technologies, it is necessary to verify the possibility of their application in the production of rubber–textile conveyor belts. This idea is based on the fact that devices currently used in additive technologies can simultaneously operate with several types of materials [17,19,20].

### 2.1. Utilization of Additive Technologies in the Production of Conveyor Belts

The production of rubber–textile conveyor belts involves the following steps:

(a) Gumming of the textile fabric;
(b) Gluing and preparing the carcass of the conveyor belt;
(c) Preparing the cover layers;
(d) Laying the cover layers and side edges, referred to as the conveyor belt assembly;
(e) Vulcanization and marking of the conveyor belts;
(f) Finishing and finalization.

The use of additive technologies can fundamentally transform the entire procedure used thus far. However, whether additive technologies provide sufficient options to produce a rubber–textile conveyor belt should be first verified.

Based on this, a multi-stage study was initiated to confirm or refute the option of manufacturing a rubber–textile conveyor belt using additive technology.

The first pre-condition of this research was to verify whether a lattice structure exists that can be used regardless of the material properties. As the conveyor belt involves a sandwich structure, it is necessary to verify whether the belt can be manufactured using existing additive technologies or whether the individual belt parts can be produced and combined using a suitable technology into a final unit representing a rubber–textile conveyor belt.

**Figure 1:**
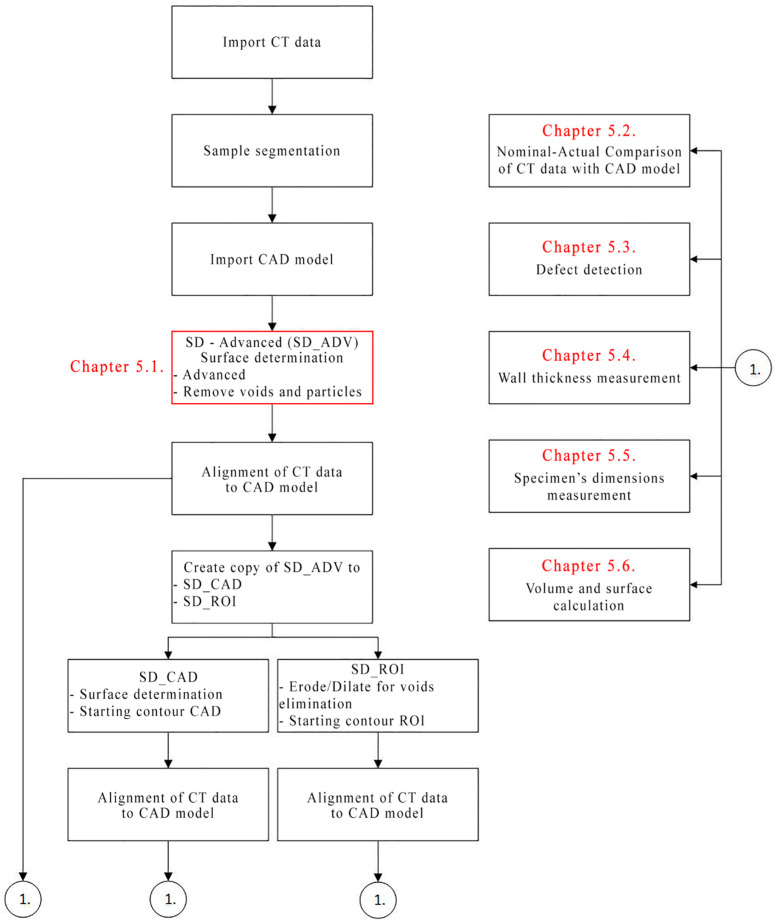
Methodology of the implemented research.

Therefore, this study focused on the possibility of producing a structure representing a conveyor belt carcass. Figure 1 depicts the proposed implementation methodology.

### 2.2. Research Methodology for Using Additive Technologies to Produce Carcasses of Rubber–Textile Conveyor Belts

A structure was identified to represent the carcass of a rubber–textile conveyor belt in the developed research methodology (Figure 1). This was conditioned by the possibility of producing it using the available additive technologies. The individual specimens obtained were assessed using non-destructive industrial computed tomography (CT) analysis; however, the material properties were omitted, as they are intended to be examined in subsequent research phases.

## 3. Theory

Rubber–textile conveyor belts are key components of continuous transport systems. Typically, they need to satisfy stringent criteria to ensure operational and technological reliability.

**Figure 2:**
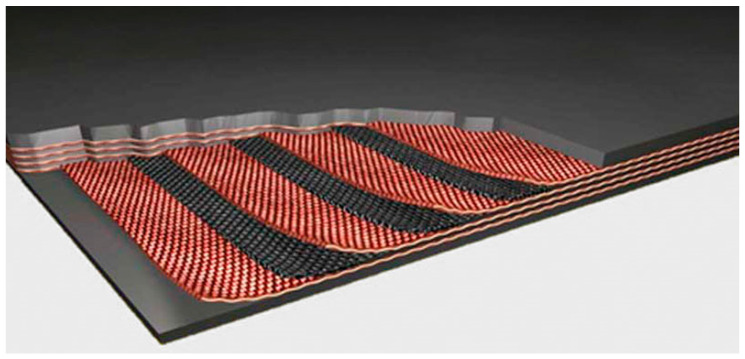
Construction of a rubber–textile conveyor belt [21].

The construction of a rubber–textile conveyor belt involves an upper covering layer, a lower covering layer, and the carcass of the conveyor belt (Figure 2).

**Figure 3:**
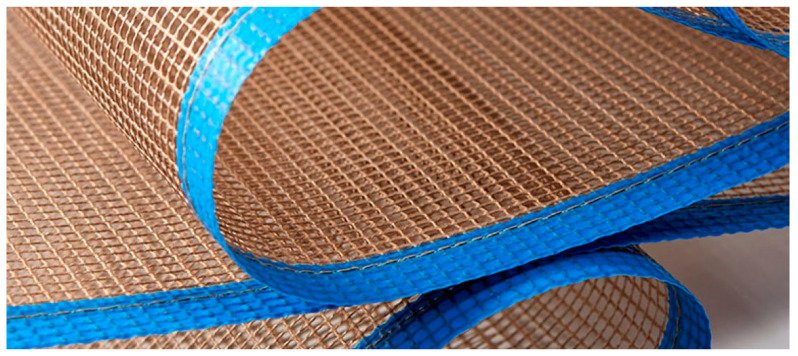
Example of a textile used to create a carcass of a rubber–textile conveyor belt [22].

The carcass of the conveyor belt ensures transmission of the pulling force. In general, the carcass is composed of several layers of textiles connected by an elastic material to create a uniform unit with the required strength and flexibility. The carcass and its upper and lower covering layers must absorb the energy generated by the materials placed on the conveyor belt. Another feature of the rubber–textile conveyor belt carcass is to ensure the pulling strength required for the movement of a loaded conveyor belt. The carcass must be sufficiently rigid for the conveyor belt to support the transported load. Rubber–textile conveyor belt carcasses can vary in thickness. The size depends on the properties of the individual textiles (Figure 3) and the operating parameters of the rubber–textile conveyor belt. A structural unit with a certain strength and elastic properties can be obtained by connecting textiles in several layers.

### Gyroid

In certain cases, the structural geometry is formed by minimum areas. Minimum areas or surfaces are porous materials obtained by densifying mathematically minimal areas into a finite spatially homogeneous thickness until a solid volume fraction is attained. The occurrence of minimal surface geometries in biological tissues, such as shells of beetles, bumblebees, and butterfly wings, confirms their use in biomedicine, including the design of bio tissues. Well-established minimum surfaces exhibit a cubic symmetry and can be created using repeated periodic cubic translations. The continuously curved Schoen-structured gyroid is the most common representative of these structures. The gyroid structure divides the structural space into two or three regions and minimizes the interfacial area. A basic building block of the entire gyroid structure comprises a complex curved surface that forms struts of circular cross-sections, generating an almost spherical hollow core. Each building block of the structure includes three points; three struts converge into each of them, and the entire gyroid occupies a cube with a specific edge length in a 3D space [23,24].

## 4. Specimen Design Methodology

**Figure 4:**
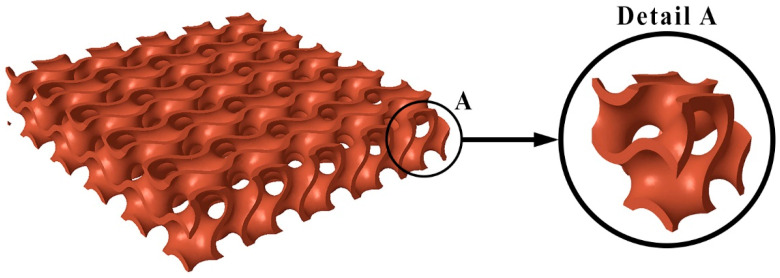
Three-dimensional (3D) model of the gyroid sample created using PTC Creo Parametric 9.

In this study, 3D models of the experimental specimens were created using the PTC Creo Parametric version 9.0 software. Figure 4 illustrates the 3D model of the specimen and a detailed view of the gyroid cell. A cuboid with dimensions of 35 mm × 35 mm × 7 mm served as the basis for creating the 3D model, wherein the volume was filled with a gyroid structure. The size of the basic gyroid cell in all three directions along the X-, Y-, and Z-axes was 7 mm. The position of the basic gyroid cell was identical in all experimental specimens; in other words, the entire cell was multiplied (patterned) in all directions of the coordinate system.

**Figure 5:**
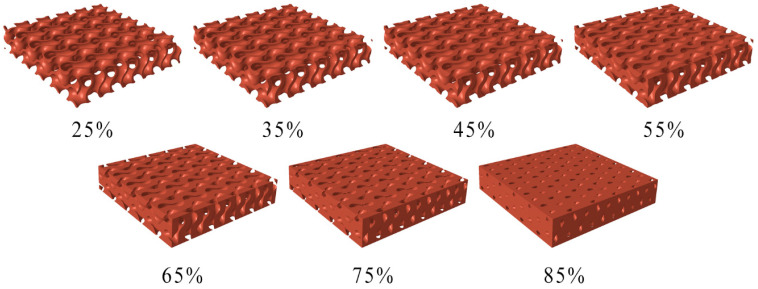
Three-dimensional (3D) models of gyroid specimens considering seven relative densities.

Seven gyroid samples with different relative densities (RD) were prepared. Relative densities of the samples were controlled by changing the wall thickness of the gyroid cell from 0.50 to 1.64 mm. Seven relative densities, RD = 25, 35, 45, 55, 65, 75, and 85%, were created. Figure 5 depicts the 3D models of individual specimens with different relative densities.

The experimental specimens were fabricated using the Original Prusa i3 MK3S+ 3D printer (Prague, Czech Republic) and material extrusion method. The experimental specimens were printed according to the printing parameters listed in Table 1. Flexfill 92A (Hulin, Czech Republic), which is flexible and elastic and exhibits suitable mechanical properties, was used to fabricate the specimens (Table 2). Table 3 summarizes the parameters of the 3D models of the experimental specimens based on which STL files were subsequently created.

## 5. Methodology of CT Scanning and Assessment

The use of standard measurement methods and devices is limited with respect to the volume structure of the designed specimens. One method of obtaining relevant information on production accuracy through non-destructive testing (NDT) is to use industrial CT. The process of obtaining and assessing CT scan data can be divided into five steps as follows:

1. Scanning the fabricated specimens: Scanning was performed using an industrial computer (Metrotom 1500; Carl Zeiss, Jena, Germany). The specimen dimensions (35 mm × 35 mm × 7 mm) enabled the simultaneous scanning of the two specimens. During scanning, the specimens were attached to a polystyrene holder and separated from each other using a polystyrene partition. A resolution of 50.880 µm was achieved during scanning.
2. Segmentation and determination (reconstruction) of the CT scan surface: The surface of the CT scan was segmented and reconstructed using VGStudio MAX 2.2 software (Volume Graphics, Heidelberg, Germany). As part of the surface-reconstruction analysis, three surface determination (SD) options were tested, including:
  - Automatic with advanced mode (SD_ADV);
  - Automatic with advanced mode and CAD starting contour (SD_CAD);
  - Automatic with advanced mode and region of interest (ROI) starting contour (SD_ROI).
3. Analysis of specimen volume, percentage material volume, and specimen surface: The volume of the specimen material and its surface area were determined based on SD.
4. Porosity analysis of the fabricated specimen: The defect detection module was used for the porosity analysis of the fabricated specimen.
5. Analysis of specimen production accuracy: The fabricated specimen was compared with a reference CAD model using a nominal–actual comparison model. The wall thickness of the specimen was measured using a distance tool.

An exact sequence of actions must be observed for analyses that depend on previously performed actions. The calculation of volume, surface area, and porosity depends on the SD. The comparison of the specimen with the CAD model and measurement of the wall thickness depended on the SD and alignment of the specimen with the CAD model. The following points and flowchart in Figure 1 describe the procedure of the individual analysis.

1. Creating SD_ADV.
2. Specimen alignment with the CAD model using manual alignment and the best-fit method.
3. Duplicate SD_ADV to SD_CAD and SD_ROI.
4. Determination of the SD_CAD surface based on the CAD model.
5. Creation of ROI from the surface in the SD_ROI scan and creation of erode/dilate for void elimination.
6. Determination of SD_ROI surface based on the created ROI.
7. Determination of porosity for SD_ADV, SD_CAD, and SD_ROI.
8. Creation of nominal–actual comparison for individual surfaces of SD_ADV, SD_CAD, and SD_ROI.
9. Comparison of deviations between SD_CAD and SD_ROI.
10. Performing a wall thickness measurement.
11. Subtraction of the volume, surface area, porosity, and cumulative part deviation values for SD_ADV, SD_CAD, and SD_ROI.
12. Measurement of sample dimensions.

### 5.1. Surface Determination of a Specimen

As the two specimens were scanned simultaneously, individual specimens were segmented using the ROI tool prior to the final surface determination. Initial reviews of the individual methodologies and analyses were conducted on specimens with a theoretical material content of 45%.

**Figure 6:**
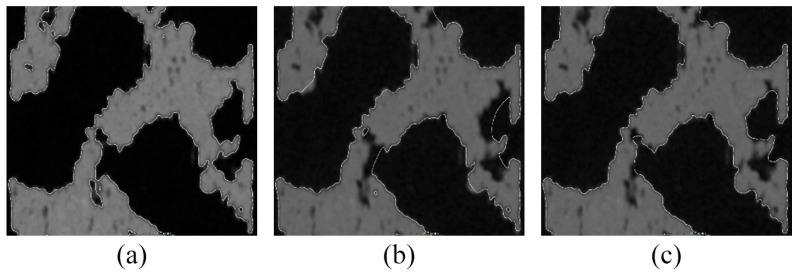
Details of surface determination: (**a**) SD_ADV, (**b**) SD_CAD, and (**c**) SD_ROI.

For basic surface determination in the SD_ADV setting, a surface search distance was set to 0.5 mm and particle removal was executed; all void settings were used to close small voids. As indicated in Figure 6a, although the surface determination directly duplicates the specimen surface, larger material voids are not closed. The SD_CAD and SD_ROI settings were used as the alternative surface determination. Before applying the SD_CAD model, the CT scan of the specimen and the CAD model were aligned (Section 5.2) because the surface of the CAD model was used as a reference.

In the surface determination with the SD_ROI setting, the ROI was created from the surface obtained from the SD_ADV setting; the erode/dilate function was used to close the larger voids. The surfaces created using SD_CAD and SD_ROI settings were distorted. This was more apparent in SD_CAD, wherein the surface was created in an area with no material or where the presence of material was ignored (Figure 6b). As the difference between the CAD model and CT scan of the specimen increased, this effect also increased. In the surface determination with the SD_ROI setting (Figure 6c), the distortion was caused by the erode/dilate function used to close the material voids. As the surface of the specimen produced using material extrusion technology was significantly layered, its use closed even small irregularities and voids on the surface of the specimen (Figure 6c).

**Figure 7:**
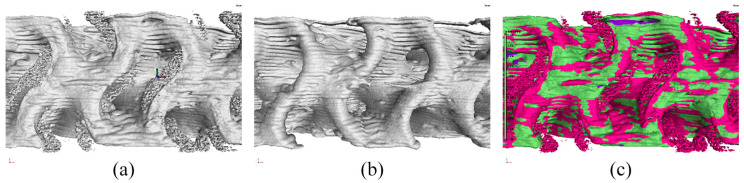
Comparison and surface determination of specimens: (**a**) SD_CAD noise, (**b**) SD_ROI noise, and (**c**) differences between the noise generated using SD_CAD and SD_ROI indicated in burgundy.

Figure 7 depicts the noise in the surfaces obtained using SD_CAD and SD_ROI settings along with their mutual comparison. In the surface determination with the SD_CAD settings, unnatural noise was generated in the corners where the material should ideally exist but was no longer present. This distorted the surface and the resulting porosity value (Figure 7a). In the surface determination with the SD_ROI setting (Figure 7b), larger deviations were observed in certain areas. Based on this information, the volume of the material in the specimen was deducted from the surface determination in the SD_ADV and SD_ROI settings to eliminate most of the voids present in SD_ADV and avoid the extent of distortion that was observed under the SD_CAD setting (Figure 7c).

#### Comparison of Individual Surface Determinations with a CAD Model

**Figure 8:**
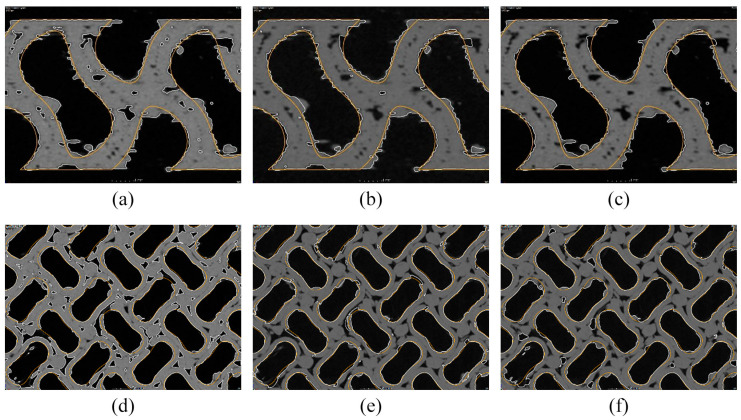
Sections comparing the surface determination with a computer-aided design (CAD) model: (**a**) and (**d**) SD_ADV; (**b**) and (**e**) SD_CAD; and (**c**) and (**f**) SD_ROI.

Each model was aligned to perform the nominal–actual comparison of individual models (SD_ADV, SD_CAD, and SD_ROI) with the CAD model. The alignment was performed in three steps. The first step was a rough alignment using the best-fit method, wherein the volume of the CAD model overlapped with that of the specimen. Owing to the structural complexity, the position of the specimen was considered but not its orientation (rotation). Rotation was performed manually in the second step. The third step involved a "fine" best fit with the option of current transformation during the rotation of the specimen. The best fit was selected based on the production technology and nature of the specimen. Figure 8 depicts the cross-sections in two views; the white line indicates the individual surface determinations in SD_ADV (Figure 8a,d), SD_CAD (Figure 8b,e), and SD_ROI (Figure 8c,f) in the scan, whereas the orange line denotes the CAD model. The figure indicates the differences between individual surface determinations with respect to the CAD model. A nominal–actual comparison was performed based on this alignment.

### 5.2. Nominal–Actual Comparison of CT Data with the CAD Model for SD_ADV, SD_CAD, and SD_ROI

**Figure 9:**
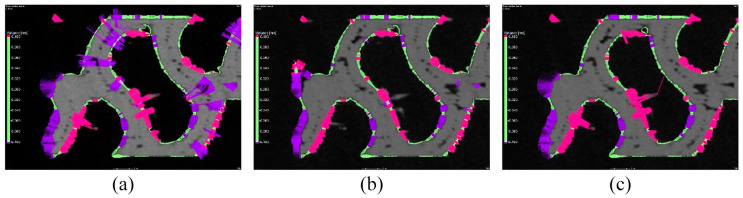
Nominal–actual comparison of individual surface determinations with the reference computer-aided design (CAD) model: (**a**) SD_ADV, (**b**) SD_CAD, and (**c**) SD_ROI.

In the nominal–actual comparison of SD_ADV and the reference CAD model (Figure 9a), significant deviations were observed in the areas of larger voids, which could not be eliminated during surface determination. In the nominal–actual comparison of SD_CAD and the reference CAD model (Figure 9b), significant deviations were observed in the areas with an artificial increase or decrease in material volume, and a considerable number of voids were closed; therefore, deviations were not assessed. In the nominal–actual comparison of SD_ROI and the reference CAD model (Figure 9c), most of the internal voids were eliminated like that observed in SD_CAD (Figure 9b). However, unlike the SD_CAD, no significant surface distortion was observed. Therefore, the nominal–actual comparison of SD_ROI resembled that of SD_ADV (Figure 9a).

**Figure 10:**
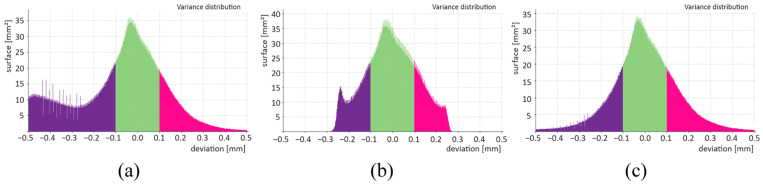
Histograms of individual nominal–actual comparisons: (**a**) SD_ADV, (**b**) SD_CAD, and (**c**) SD_ROI.

Histograms of surface deviations were used for SD_ADV, SD_CAD, and SD_ROI determination and compared with the CAD model (Figure 10). The impact of surface determination of the specimen when compared with the CAD model was apparent. While several deviations with a negative value in unclosed voids were observed in SD_ADV, a significant "cutting" of deviations caused by surface deformation was observed in SD_CAD. The SD_ROI histogram benefited from the elimination of small voids in the material (compared with SD_ADV) and minor surface deformations (compared with SD_CAD). The maximum deviation in 90% of the surface was determined from a cumulative histogram; in other words, the deviation was less than or equal to a certain value in 90% of the surface.

**Figure 11:**
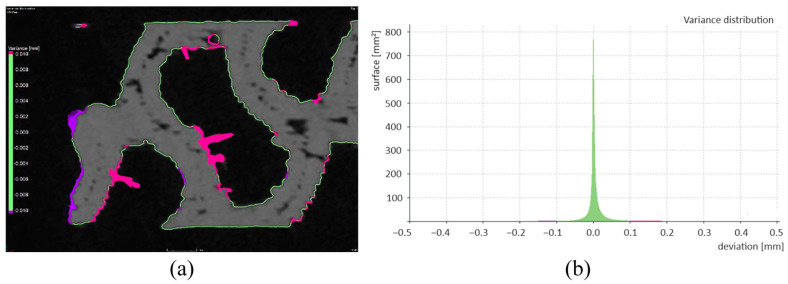
Nominal–actual comparison of SD_ROI and SD_CAD with (**a**) the display of deviations and (**b**) the histogram of deviations.

The areas where the created surfaces of SD_CAD and SD_ROI differed the most were analyzed; their mutual nominal–actual comparison was performed in a sample with a material volume of 45% (Figure 11a). SD_CAD and SD_ ROI were used as the nominal and actual objects, respectively. In this case, the impact of noise on SD_CAD was eliminated because the noise was absent in SD_ROI. The value of deviations, indicated in green, was changed to ±0.01 mm; Figure 11b illustrates their distribution in a histogram. With increasing deviations, the comparison ceased to exhibit informative value; therefore, further information on this is not presented in this paper.

### 5.3. Defect Detection in SD_ ADV, SD_CAD, and SD_ROI

**Figure 12:**
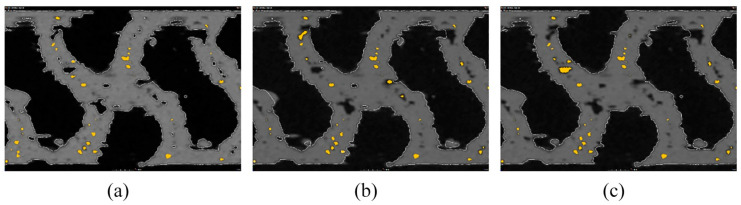
Differences in porosity assessment with respect to (**a**) SD_ADV, (**b**) SD_CAD, and (**c**) SD_ROI.

For individual surface determination, pore analysis was performed with the minimum pore size set to eight voxels and the maximum pore diameter set to 100 mm. The percentage of the volume of pores (cavities and cracks) in the total volume of material created based on surface determination is called porosity. Typically, surface determination (ISO surface value), the pore shape, and the surface itself are known to significantly influence the void size. Figure 12 depicts the porosities of individual surfaces. In comparison with SD_ADV (Figure 12a), the determination of the SD_CAD surface (Figure 12b) and SD_ROI surface (Figure 12c) affected the surface itself (explained in Section 5.1).

### 5.4. Wall-Thickness Measurement

**Figure 13:**
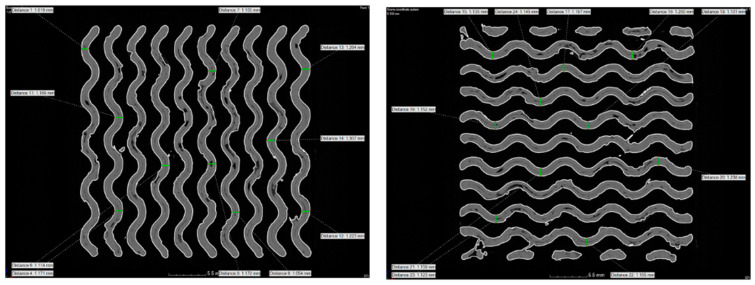
Measurement locations of the wall thickness in a specimen with 45% material volume in two mutually perpendicular layers.

The wall thickness of the specimen was assessed as part of the analysis. The measurements were performed in two mutually perpendicular layers with 10 locations measured in each layer. The measurement locations were designed to cover the entire cross-sectional area of the specimen and selected from locations without visible defects (visibly reduced or increased wall thickness). The arithmetic means and standard deviations were calculated from the measured values. Figure 13 illustrates the wall thickness measurement locations for a specimen with 45% material volume (green lines). For comparison, the wall thicknesses were measured at the same locations as those in the reference CAD models.

### 5.5. Measurement of Specimen Dimensions

**Figure 14:**
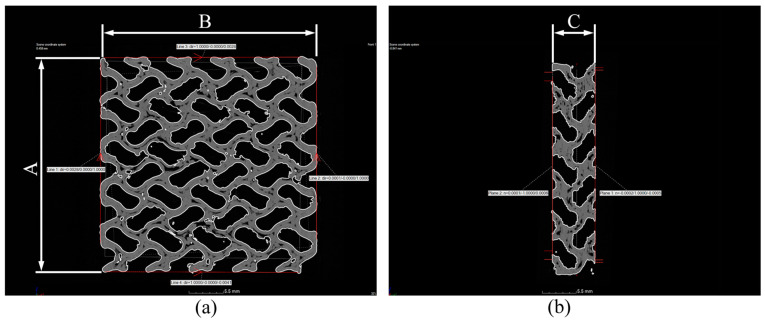
Features created for measuring the dimensions of specimens. (**a**) Measuring the base dimensions and (**b**) the height. A,B,C—sample dimensions.

The outer dimensions of the specimens were measured to determine the shrinkage and increase in their dimensions. The corners were excluded as they tended to deform. To measure the base dimensions (dimensions A and B)*,* lines 1–4 were generated in a selected cross-section in the center of the specimen, and the mid-perpendicular distances between lines 1 and 2 and lines 3 and 4 were measured in the selected plane. The height (dimension C) was assessed as the mid-perpendicular distance between the created planes (planes 1 and 2). The measurements were performed on the SD_ROI surface; Figure 14 illustrates these features.

### 5.6. Volume and Surface Calculation

The values of the object (material) volume and surface area were obtained based on the volume and region properties. The calculation of volume, surface area, and porosity depended on the surface determination. In the case of SD_ROI, the surface determination described the surface of the sample and included pores of all sizes, whereas SD_ADV predominantly included small-sized pores (Figure 6).

## 6. Results and Discussion

Table 4, Table 5, Table 6, Table 7, Table 8, Table 9 and Table 10 list the measured and assessed values of the specimen volume, surface area, porosity, and cumulative deviation from the CAD model for individual surface determinations. The dimensions of the manufactured specimens affected their volume and surface.

With an increasing percentage of material volume in the specimen, the printed shape exhibited increasing deviations from the nominal CAD model. Owing to the different philosophies of surface creation, the deviations from the CAD model were the lowest in the case of SD_CAD.

The wall thicknesses of all specimens were evaluated during surface determination using the SD_ROI settings at 20 locations on each specimen. The edges of the specimens were excluded from the measurements to ensure that the results remained unaffected. The obtained values were verified using the Shapiro–Wilk normality test and Grubbs’ test for outliers (Table 11). The results exhibited a normal measurement distribution, and the individual measurement sets did not contain outliers. The wall thickness measurements indicated the smallest differences between gyroid_45 and gyroid_55.

Table 12 presents the comparison of the theoretical volume of the specimen obtained from the CAD model (*V_T_*) and the actual volume obtained from the CT data (*V_R_*) to determine the SD_ADV surface. The percentage change in volume was calculated using Equation (1) as follows:

(1) $$∆=\frac{{(V}_{R}-V_{T})}{V_{T}}\cdot 100[\%].$$

The results indicated that the percentage change in the volume of the fabricated specimens compared with the CAD reference was in the range of 0.68 to 8.6%.

Table 13 presents a comparison of the theoretical surface areas of the specimen obtained from the CAD model (*A_T_*) and the actual surface area obtained from the CT data (*A_R_*) for the SD_ROI surface determination. The percentage change in surface area was calculated using Equation (2) as follows:

(2) $${∆}_{A}=\frac{{(A}_{R}-A_{T})}{A_{T}}\cdot 100[\%]$$

The results indicated that the percentage change in the surface area of the fabricated specimens significantly varied in comparison with that of the CAD model. However, the decrease in value was attributed to the increasing volume of the material.

Finally, the external dimensions of the specimens were measured. The nominal dimensions of the reference specimen were 35 mm × 35 mm × 7 mm and the reference area was 1225 mm^2^. The results indicated that the difference between the actual and theoretical areas decreased as the percentage of material increased. Table 14 summarizes the obtained results.

## 7. Conclusions

Additive manufacturing enabled a wider application of various three-dimensional parts in practice, to lighten the products, while also maintaining the required mechanical properties. One of the most frequently used structures is the gyroid and various areas of its use are being investigated. Several authors have addressed the influence of gyroid parameters [25,26] on the mechanical properties of a designed structure [27,28,29,30] and on dimensional analysis depending on production technology and the material used [31,32,33]. This paper focuses on specimens’ metrological analysis with different relative densities manufactured using material extrusion technology from the Flexfill 92A material with the intent to provide methodological instructions for industrial computer tomography use in the assessment of objects produced by additive manufacturing. Metrological analysis determines the effect of surface determination on specimen surface reconstruction, dimensional analysis, and porosity of manufactured specimens. Surface determination significantly affects not only surface reconstruction but also most analyses into which it enters directly (e.g., defect detection) or indirectly (dimensional analysis). Three methods of surface determination were compared in the paper. The SD_ADV method best copied the specimens’ surface, but did not close the larger material pores; the SD_CAD method closed the cavities but showed larger differences between the CAD model and the specimens’ deformations and surface noise on the samples; and the SD_ROI method closed the pores in the material but also triggered small surface irregularities resulting from the production technology. Based on the results, the use of SD_ROI is the most suitable surface determination method for gyroid structure and material extrusion.

The dimensional analysis of specimens consisted of measuring their external dimensions, the structure’s wall thickness, and the nominal–actual comparison with the reference CAD model. The results show that with increasing relative density, the deviations of the external dimensions of specimens (expressed as a percentage change in area A) decreased. The wall thickness analysis shows the smallest deviation at 45% relative density, while it increases toward greater and lesser relative density. In nominal–actual comparison, the smallest cumulative deviation is at 90% for 25% relative density; deviations for other specimens do not show significant differences. The results of the volume and surface assessments of the specimens show that all specimens have a smaller volume compared with the theoretical values; the specimen surface was larger compared with the theoretical value in all specimens, which is given by the production technology triggering surface irregularities. The pore analysis shows that with the wall thickness increasing, the percentage of pores in the samples also increases. This is due to a small relative density because, in the case of a small wall thickness, there is less space for pore formation than with a larger pore density. The results of the specimen complex analysis can be used to control and optimize the manufacturing process in dimensional accuracy, the porosity of manufactured specimens, and for checking specimens in mechanical tests. The obtained point clouds can serve as an input to simulations focused on the structures’ mechanical response, while, unlike theoretical models, they also contain all defects and inaccuracies resulting from manufacturing [34,35,36].

This paper was written as part of research aimed at changing the method of manufacturing the textile carcass of rubber–textile conveyor belts. This research was based on the idea of using an additive approach. The idea of applying a new idea to manufacturing conveyor belt frames is based on knowledge development in additive technologies in terms of materials, technologies, and hardware. Based on the mentioned facts, a hypothesis was formulated, which is currently in the research stage. This study also implies a search for a suitable structure, which will be further researched, to determine the requirements for the application of conveyor belts. Within the mentioned research, the gyroid structure was also investigated, among other things. The contribution of this paper is a proposal for the assessment methodology of “gyroid” specimens produced using material extrusion technology, its verification, and the creation of recommendations to assess specimens using CT technology. When reviewing the documents, there was no paper dealing with the methodology of assessing different types of structures using CT. The individual contributions presented only research results but did not address different settings’ effects on the assessed parameters.

## Figures and Tables

**Table 1:** Printing parameters for fabricating the experimental specimens.

Nozzle temperature	240 °C
Bed temperature	50 °C
First layer speed	20 mm.s^−1^
Infill speed	70 mm.s^−1^

**Table 2:** Properties of the Flexfill 92A material.

Material density	1.20 g.cm^−3^
Filament diameter	1.75 mm
Tensile strength	49 MPa
Tensile stress	7.50 MPa
Elongation at break	600%
Tear strength	85 kN.m^−1^

**Table 3:** Parameters of the 3D models of the experimental specimens.

Volume of the Entire Sample (mm^3^)	Sample Name	Cell Size (mm)	Relative Density (%)	Wall Thickness (mm)	Sample Volume (mm^3^)
8575.000	gyroid_25	7	25.080	0.500	2150.770
	gyroid_35		35.230	0.700	3020.760
	gyroid_45		45.490	0.900	3900.990
	gyroid_55		55.410	1.090	4751.550
	gyroid_65		65.510	1.280	5617.460
	gyroid_75		75.260	1.460	6453.900
	gyroid_85		85.620	1.640	7341.530

**Table 4:** Measured and reviewed values of the specimen for 25% relative density.

Gyroid_25	V (mm^3^)	S (mm^2^)	Porosity (%)	Cumulative Deviation at 90%
SD_ADV	2103.880	9723.980	0.050	0.217
SD_CAD	2112.140	10,005.100	0.050	0.094
SD_ROI	2095.560	9433.34	0.050	0.209

**Table 5:** Measured and reviewed values of the specimen for 35% relative density.

Gyroid_35	V (mm^3^)	S (mm^2^)	Porosity (%)	Cumulative Deviation at 90%
SD_ADV	3096.830	10,294.570	0.970	0.255
SD_CAD	3029.520	10,527.770	0.640	0.096
SD_ROI	3097.000	9517.900	0.680	0.239

**Table 6:** Measured and reviewed values of the specimen for 45% relative density.

Gyroid_45	V (mm^3^)	S (mm^2^)	Porosity (%)	Cumulative Deviation at 90%
SD_ADV	3584.250	12,679.430	1.270	0.397
SD_CAD	3788.900	10,637.470	1.580	0.206
SD_ROI	3810.630	9644.390	1.740	0.240

**Table 7:** Measured and reviewed values of the specimen for 55% relative density.

Gyroid_55	V (mm^3^)	S (mm^2^)	Porosity (%)	Cumulative Deviation at 90%
SD_ADV	4342.750	14,166.270	1.610	0.388
SD_CAD	4649.900	10,841.910	2.560	0.204
SD_ROI	4637.780	10,243.940	2.970	0.245

**Table 8:** Measured and reviewed values of the specimen for 65% relative density.

Gyroid_65	V (mm^3^)	S (mm^2^)	Porosity (%)	Cumulative Deviation at 90%
SD_ADV	5540.730	11,734.600	3.600	0.602
SD_CAD	5627.270	10,840.770	3.950	0.096
SD_ROI	5739.340	9055.880	4.060	0.285

**Table 9:** Measured and reviewed values of the specimen for 75% relative density.

Gyroid_75	V (mm^3^)	S (mm^2^)	Porosity (%)	Cumulative Deviation at 90%
SD_ADV	6762.960	9615.530	3.660	0.454
SD_CAD	6282.400	11,692.600	3.650	0.240
SD_ROI	6879.870	8147.640	3.560	0.460

**Table 10:** Measured and reviewed values of the specimen for 85% relative density.

Gyroid_85	V (mm^3^)	S (mm^2^)	Porosity (%)	Cumulative Deviation at 90%
SD_ADV	7291.850	11,776.700	5.000	0.346
SD_CAD	7336.000	5999.370	5.300	0.094
SD_ROI	7553.260	7743.650	6.080	0.290

**Table 11:** Measurement of wall thicknesses in all specimens.

Sample Name	Nominal Value + SD (mm)	Diameter + SD (mm)	Percentage Change in Average Values (%)
gyroid_25	0.630 ± 0.010	0.570 ± 0.052	−9.524
gyroid_35	0.845 ± 0.073	0.875 ± 0.017	3.550
gyroid_45	1.159 ± 0.006	1.155 ± 0.069	−0.345
gyroid_55	1.409 ± 0.042	1.417 ± 0.081	0.568
gyroid_65	1.674 ± 0.010	1.805 ± 0.125	7.826
gyroid_75	2.019 ± 0.032	2.261 ± 0.117	11.986
gyroid_85	2.400 ± 0.008	2.628 ± 0.174	9.500

**Table 12:** Comparison of the theoretical volume of the specimens obtained from the CAD model (*V_T_*) and the actual volume obtained from the CT data (*V_R_*) for surface determination in SD_ROI.

Sample Name	Relative Density (%)	Theoretical Specimen Volume *V_T_* (mm^3^)	Actual Specimen Volume *V_R_* (mm^3^)	Percentage Change in Volume (%)
gyroid_25	25.080	2150.770	2103.880	−2.180
gyroid_35	35.230	3020.760	3096.830	2.520
gyroid_45	45.490	3900.990	3584.250	−8.120
gyroid_55	55.410	4751.550	4342.750	−8.600
gyroid_65	65.510	5617.460	5540.730	−1.370
gyroid_75	75.260	6453.900	6762.960	4.790
gyroid_85	85.620	7341.530	7291.85	−0.680

**Table 13:** Comparison of the theoretical surface area of the specimen obtained from the CAD model (*A_T_*) and the actual surface area obtained from CT data (*A_R_*) during the surface determination based on SD_ROI settings.

Sample Name	Theoretical Surface Area of the Specimen *A_T_* (mm^2^)	Actual Surface Area of the Specimen *A_R_* (mm^2^)	Percentage Change in Surface Area (%)
gyroid_25	8090.080	9433.340	16.600
gyroid_35	8183.700	9517.900	16.300
gyroid_45	8202.420	9644.390	17.580
gyroid_55	8173.850	10,243.940	25.330
gyroid_65	8064.180	9055.880	12.300
gyroid_75	7852.280	8147.640	3.760
gyroid_85	7285.030	7743.650	6.300

**Table 14:** Measurement of specimen dimensions.

Sample Name	A (mm)	B (mm)	C (mm)	Area A × B (mm^2^)	Percentage Change in Area A × B (%)
gyroid_25	34.642	34.074	6.932	1180.392	−3.641
gyroid_35	34.548	34.263	6.893	1183.718	−3.370
gyroid_45	34.535	34.581	6.925	1194.255	−2.510
gyroid_55	34.682	34.557	6.946	1198.506	−2.163
gyroid_65	34.627	34.635	6.946	1199.306	−2.097
gyroid_75	34.706	34.646	6.909	1202.424	−1.843
gyroid_85	34.681	34.649	6.937	1201.662	−1.905

## Data Availability

Data are contained within the article.
